# Epidemiology of ESBL-producing *Escherichia coli* from repeated prevalence studies over 11 years in a long-term-care facility

**DOI:** 10.1186/s13756-021-01013-7

**Published:** 2021-10-19

**Authors:** Romain Martischang, Patrice François, Abdessalam Cherkaoui, Nadia Gaïa, Gesuele Renzi, Americo Agostinho, Monica Perez, Christophe E. Graf, Stephan Harbarth

**Affiliations:** 1 Infection Control Programme and WHO Collaborating Centre on Patient Safety, University of Geneva Hospitals and Faculty of Medicine, Geneva, Switzerland; 2 Genomic Research Laboratory, Geneva University Hospitals, Geneva, Switzerland; 3 Bacteriology Laboratory, Geneva University Hospitals, Geneva, Switzerland; 4 Department of Rehabilitation and Geriatrics, Geneva University Hospitals, Geneva, Switzerland

**Keywords:** *Escherichia coli*, Molecular epidemiology, PCR, ST131, Antimicrobial resistance, Fluoroquinolone resistance, Intestinal colonization, Surveillance, Long-term care facility

## Abstract

**Background:**

*Escherichia coli* sequence type (ST) 131 H30 is an emerging multidrug resistant subclone, known to spread and cause outbreaks in long-term care facilities (LTCFs).

**Objectives and methods:**

From 2010 through 2020, we performed 11 yearly surveillance studies for determining the prevalence of digestive carriage of ESBL-producing *E. coli* (ESBL-EC) among residents in a university-affiliated LCTF. Sequencing and genotyping of selected isolates were performed to characterize temporal trends in the prevalence and epidemic potential of ESBL-EC subclones, and for evaluating a potential rebound effect following discontinuation of contact precautions for ESBL-EC carriers in January 2019.

**Results:**

This study included 2′403 LTCF residents, with 252 (10.5%) positive for ESBL-EC. Among the 236 ESBL-EC isolates available for typing, 58.0% belonged to the ST131 lineage, including 94/137 (68.6%) ST131 H30 isolates. An increasing yearly prevalence was observed for ESBL-EC (from 4.6 to 9.4%; *p* = 0.11), but not for the ST131 H30 subclone, which peaked in 2015 and declined thereafter. Multiple previously unnoticed ESBL-EC outbreaks occurred in the LTCF. Since 2018, we noted the clonal expansion of a rare ST131 H89 subclone (O16:H5) harboring CTX-M-14 and CTX-M-24. No rebound effect was observed in ESBL-EC prevalence nor in the different subclones following discontinuation of contact precautions for ESBL-EC carriers since 2019.

**Conclusion:**

Clonal fluctuation was observed for ST131 H30 ESBL-EC with a current decline in prevalence. Surveillance should include the evolution of ST131 non-H30 subclones, which may spread in LTCFs. Our findings suggest that discontinuation of contact precautions for ESBL-EC carriers in LTCFs may be safely implemented, in support of European recommendations to limit ESBL-producing Enterobacteriaceae control measures in endemic settings to non-*E. coli*.

**Supplementary Information:**

The online version contains supplementary material available at 10.1186/s13756-021-01013-7.

## Introduction

The global spread of extended-spectrum beta-lactamase producing *Escherichia coli* (ESBL-EC) is driven by the emergence of successful clones such as *E. coli* ST131, particularly transmissible in long-term care facilities (LTCFs) [1, 2]. For instance, between 1996 and 2014, an increase of ESBL-EC was noticed in French LTCFs, reflecting clonal spread, with a 18.1% prevalence of ST131 clones [3, 4]. In Swiss nursing homes, the proportion of ESBL-EC increased from 5 to 22% between 2007 and 2017 [5].

The increasing prevalence of *E. coli* ST131 among LTCFs is mostly explained by the clonal expansion of emerging multi-resistant clades of ESBL-EC [6], responsible for silent clusters among residents in LTCFs [7], including the fluoroquinolone-resistant clades C1 (C1/H30-R) and C2 (C2/H30-Rx) [8]. The reasons behind this apparent success remain controversial, but recent genomic and proteomic studies suggest that an improved anaerobic metabolism, as well as other human colonization and virulence factors helped this clone to outcompete the gut commensal niche [9–11], with consecutive prolonged colonization [12]. This lineage particularly fostered the community spread of CTX-M, by the maintenance of clade-restricted multi-drug resistant plasmids [13]. A nested cohort study of a large clinical trial recently observed the dominance of C1/H30-R ESBL-EC in participating European LTCFs [14]. In that study, 49% (16/33) of all ESBL-EC ST131 carriers in Geneva were positive for C1/H30-R, compared to 20–39% in the 3 other centers outside Switzerland.

Considering the excess mortality and hospital stay associated with third-generation-cephalosporin-resistant *E. coli* [15, 16], the epidemic potential of these ESBL-EC clades represents an infection control challenge in LTCFs, in particular in institutions without contact precautions for ESBL-EC carriers [17]. Effectively, many LTCFs around the world have discontinued contact precautions for ESBL-EC carriers, in light of recent studies on low nosocomial ESBL-EC transmission rates and endemic community carriage [17].

In our university-affiliated LTCF, yearly prevalence surveys were conducted from 2010 to 2020 as routine surveillance strategy to monitor the epidemiology of ESBL-producing Enterobacterales (ESBL-PE). In the present study, we sought to (i) characterize the temporal trends in the prevalence of ESBL-EC subclones among LTCF residents; (ii) combine epidemiological information with sequencing approaches to estimate the epidemic potential of emergent ESBL-EC subclones; and (iii), determine a potential rebound effect after de-implementation of contact precautions for carriers of these subclones.

## Methods

### Design and setting

This 11-year retrospective study was constituted by yearly prevalence surveys from 2010 through 2020, performed during January–February of each year, among all LCTF residents. Eight long-term care wards from a same geographical site, representing 216 beds were included. From 2018 onwards, we added four long-term care wards from a second site, representing 73 beds.

### Outcomes

The primary outcome included the overall prevalence of ESBL-EC carriage and abundance of different subclones across years, defined as the total number of positive cases per 100 screened residents. Secondary outcomes included the overall prevalence of any ESBL-PE, the number of clusters (i.e. at least two residents sharing a ST131 H30, ST131 non-H30, and non-ST131 strain in the same ward the same year), the prevalence of subclones in the wards concerned by these clusters, and the proportion of clonally related strains among these clusters. Clonal relatedness was defined based on genomes, using a threshold in the pairwise distance of ≤ 10 SNP differences, as suggested elsewhere [18].

### Infection control practices

In addition to standard precautions, until December 2018, all identified ESBL-PE carriers were placed under contact precautions, including gloves, hydrophobic coats, and, whenever possible, isolation in single-bed rooms. Contact precautions were abandoned for ESBL-EC from January 2019 onwards, with simultaneous reinforcement of standard precautions using routine observation and feedback by infection control nurses, in particular hand hygiene.

### Health-related data

Epidemiological information was prospectively collected for each participant during the surveys, including ward location, admission date, date of sampling, previous positive cultures, age, and gender. We collected yearly hand hygiene adherence of healthcare workers in LTCFs from 2014 to 2020 according to WHO methods, as well as the length of stay of all residents in the concerned wards during January and February, from 2010 to 2020.

### Microbiological methods

Rectal swabs (E-swab, Copan) or stool cultures were collected for all participants, and processed using selective chromogenic agar (ChromID ESBL; bioMérieux). All colonies that met the expected chromogenic features provided in the manufacturers' specifications were identified by matrix-assisted laser desorption ionization–time of flight (MALDI-TOF) mass spectrometry (Bruker Daltonics, Bremen, Germany) and the antibiotic susceptibility profiles of each isolate was determined by the disk diffusion method using EUCAST breakpoints and recommendations [19]. Double-disk synergy tests (DDST20 and DDST30) were used for ESBL confirmation, ensuring a high sensitivity and specificity for ESBL-PE detection [20]. Assessment by ESBL + AmpC Screen Kit 98008 (Rosco Diagnostica, Denmark) was also performed to identify the partially derepressed AmpC whenever the results of the DDST20 and cefoxitin tests were not conclusive.

### Molecular typing

Allelic discrimination qPCR assays were performed on all newly detected ESBL-EC to ascertain ST131 lineages and H30 subtypes. For known carriers, we only retained the first ESBL-EC strain if isolated in the prior 12 months. Five single nucleotide polymorphism assays targeting specific positions in 2 genes used for MLST and constituting a unique signature of ST131 were selected and validated against a collection of > 90 sequenced strains from highly diverse genetic backgrounds, as previously described [21]. The 6th assay was created from an existing in silico PCR and targets H30 through a coding point mutation in *FimH* sequence. This allelic discrimination TaqMan™ system has been validated on a collection of approximately 100 clinical strains isolated from bloodstream infections in 2015 in our institution (not published). Subclades ST131 H30 were then defined according to fluoroquinolone resistance (C1/H30-R) and additional presence of the *bla* gene CTX-M-15 (C2/H30-Rx).

### Sequencing and assembly

Candidate strains for sequencing included ST131 H30 strains observed in large clusters since 2010 (with at least 4 positive cases from the same ward), and in all clusters since 2018 (with at least 2 positive ward mates). Moreover, all non-ST131 and ST131 non-H30 isolates from 2018 onwards were sequenced. Only the first isolate per patient and one morphotype per plate were considered for typing and sequencing. Purified genomic DNA (DNeasy, Qiagen) of selected isolates was sequenced using Illumina HiSeq2500 device using 100 base pairs (bp) paired-end reads and bar codes strategy according to the Nextera XT kit (Illumina), following the manufacturer's recommendations. Read quality was assessed with the Fastqc program (http://www.bioinformatics.babraham.ac.uk/projects/fastqc/) and filtered using the FastqMcf program (Ea-utils; http://code.google.com/p/ea-utils). Genome assembly was performed using Spades assembler v 3.12.0. Assembled genomes were submitted individually to the Center for Genomic Epidemiology (https://cge.cbs.dtu.dk/) for confirmation of serotypes by using FimTyper 1.0 and SerotypeFinder 2.0.

### Core genome multi-locus sequence typing target genes

The task template “*E. coli* cgMLST v1.0” was used in a multi-locus sequence typing (cgMLST) scheme with Ridom SeqSphere + software version 5 (Ridom GmbH, Germany) using default settings. The final cgMLST scheme consisted of 2′513 genes covering roughly 45% of the genomic sequence of *E. coli*. From each isolate, the complete sequence of each gene was analyzed according to the cgMLST scheme and a numerical allele type was assigned to that given locus. The allelic profile was therefore determined by combining alleles of all cgMLST loci for each strain. A minimum spanning tree (MST) was inferred by neighbor joining method on the allelic profiles. The remaining genes were used for pairwise-comparisons.

### Statistical methods

Proportions were compared using χ^2^ tests, or two-tailed Fisher’s exact test when appropriate. The prevalence curve was segmented based on seeming inflection points for statistical comparison, as defined elsewhere [22]. A chi-square test for linear trend across these segments assessed prevalence shifts over time [23]. Genetic diversity was estimated using the number of ST divided by the number of strains sequenced. All analyses were conducted using R.4.0, including the package “lme4”.

## Results

**Fig. 1:**
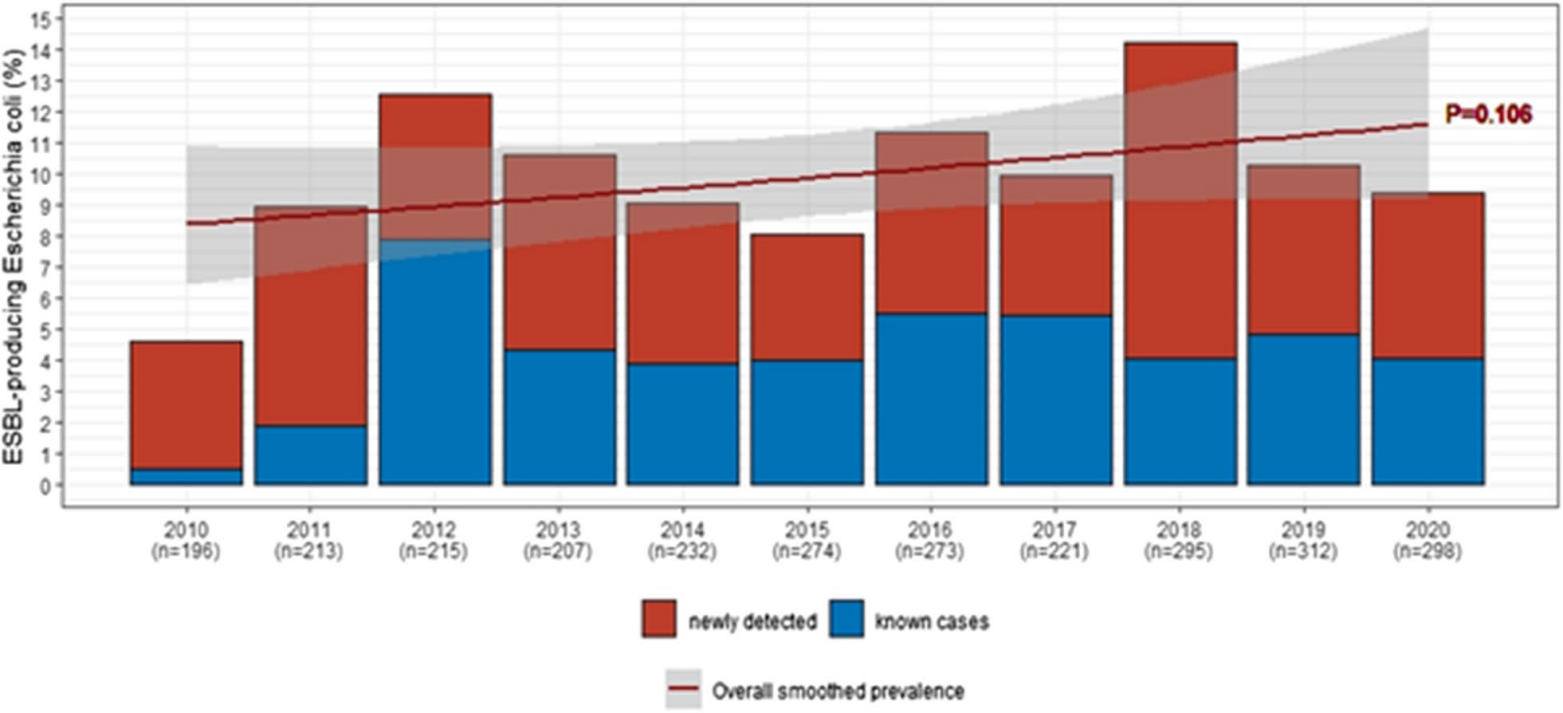
Prevalence of ESBL-producing *Escherichia coli* carriage among all residents of a university-affiliated long-term care facility from 2010 to 2020, before and after de-implementation of contact precautions in January 2019, and stratified between previously known and newly detected carriers

**Fig. 2:**
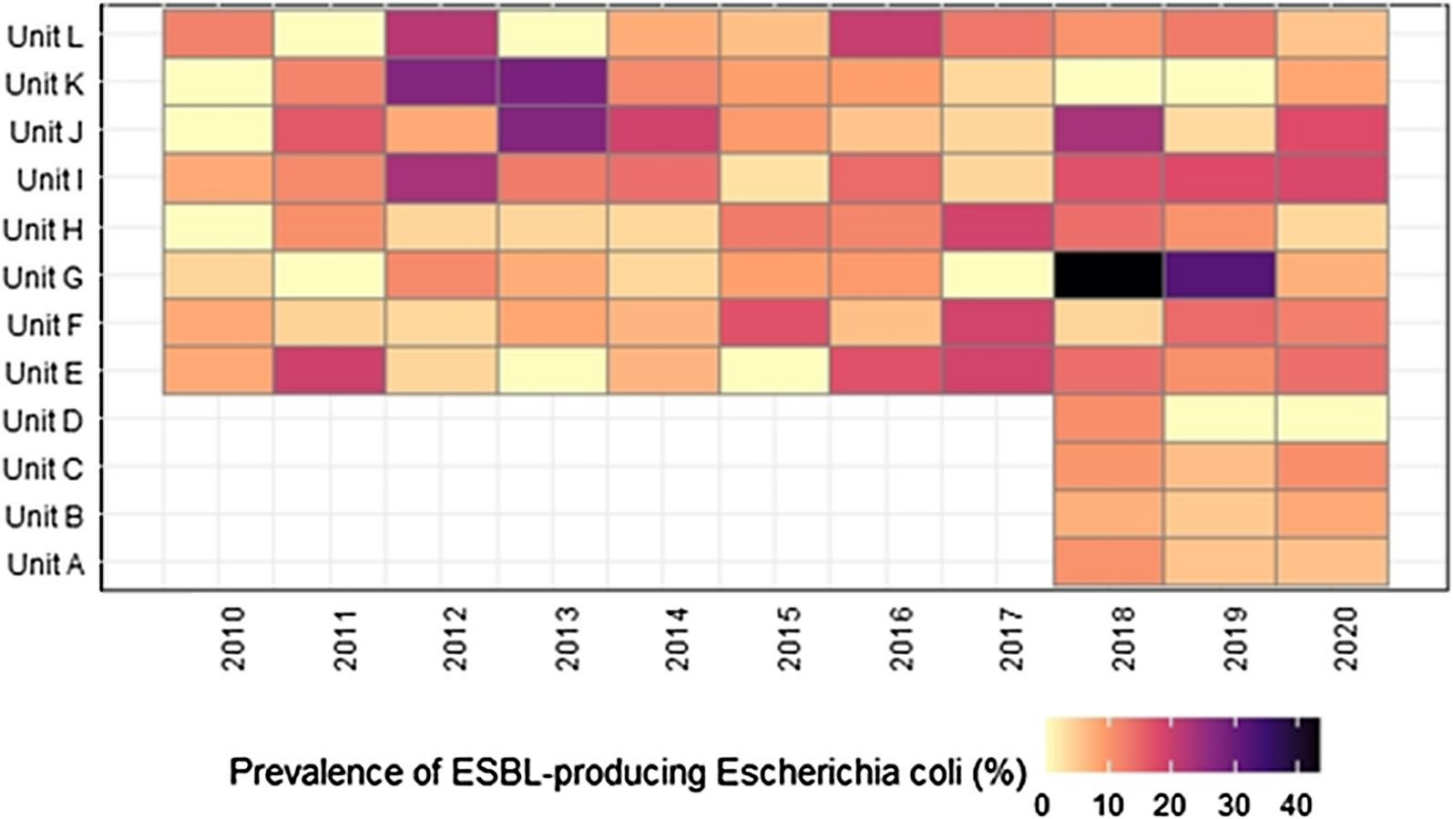
Yearly prevalence and clustering of ESBL-producing *Escherichia coli* carriers within 12 wards of a university-affiliated long-term care facility, Geneva (2010 to 2020). From 2018 onwards, 4 long-term care wards were added from a separate facility

From January 2010 through February 2020, 11 yearly cross-sectional surveys of ESBL-PE carriage included 2′403 LTCF residents, with a median age of 83 years (IQR 75–89), and 61.4% of women. Yearly hand hygiene adherence improved from 72 to 77% from 2016 to 2020 (Additional file 1: Figure S1). The median length of stay of patients hospitalized in January and February from 2010 to 2020 decreased from 138.0 days (IQR 60.9–321.0) to 33.8 days (18.0–74.4.0). The total prevalence of any ESBL-PE carriage was 13.3% (n = 319) and doubled from 7.1% to 13.8% over 10 years (*p* = 0.04). Among ESBL-PE positive patients, 79.0% (n = 252) and 18.8% (n = 60) were respectively colonized with *E. coli* and *Klebsiella pneumoniae*. Over the study period, ESBL-EC prevalence increased from 4.6% to 9.4% (*p* = 0.11), with a peak of 14.2% in 2018 (Fig. 1). We observed an increase of prevalent (previously known) cases from 11.1% to 43.0% of ESBL-EC from 2010 to 2020, with a stable proportion of incident (newly identified) cases. Of note, this increase was partly driven by nosocomial clusters throughout multiple wards in 2012, 2013, 2018, and 2019 (Fig. 2).

**Fig. 3:**
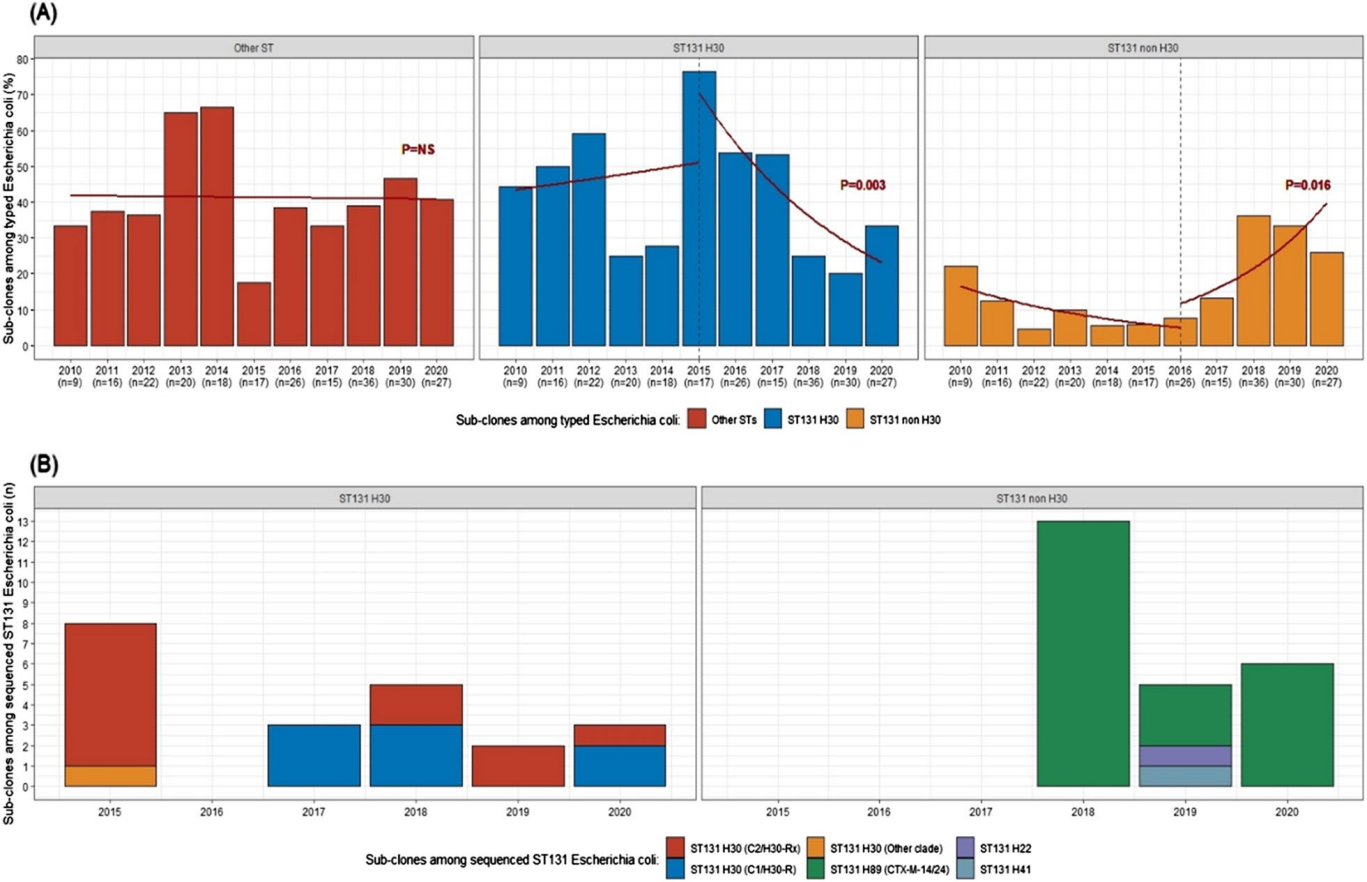
Prevalence of the different subclones among typed ESBL-producing *Escherichia coli* (ESBL-EC), and number of clades identified among sequenced ESBL-EC from residents of a university-affiliated long-term care facility from 2010 to 2020. **A** Subclones of typed ESBL-EC. Test for linear trends over segmented or continuous periods are indicated for the three subclones. Number of isolates per year are shown below the x-axis. **B** Subclones & clades of sequenced ESBL-EC ST131 since 2015

Overall, 58.0% (137/236) of typed ESBL-EC isolates belonged to the ST131 lineage, with 68.6% (94/137) positive for ST131 H30. The prevalence of this subclone remained stable until 2015 (Fig. 3A), with a subsequent downward slope deflection from 2015 to 2020 (76.5% to 33.3%, *p* < 0.003). No rebound effect was recorded neither for ESBL-EC, nor specifically for ESBL-EC ST131 H30 following de-implementation of contact precautions for ESBL-EC carriers in January 2019. In contrast, we observed an increase of ST131 non-H30 subtypes from 2016 to 2020 (*p* = 0.016), which peaked in 2018 (Fig. 3A). In total, 82 of 236 (34.7%) typed ESBL-EC were sequenced, including 11 ST131 H30 strains from large nosocomial clusters in 2010–2017, 10 ST131 H30 strains from clusters in 2018–2020, as well as 24 ST131 non-H30 and 37 non-ST131 strains, isolated since 2018 (Additional file 1: Table S1). Among ST131 H30 isolates, 12 belonged to the clade C2/H30-Rx (57.1%), and 8 to the clade C1/H30-R (38.1%; Fig. 3B). Of note, whereas the majority of C2/H30-Rx strains (58.3%) were detected in 2015, C1/H30-R strains were only detected from 2017 onwards. Among ST131 non-H30, we observed the emergence and expansion of 22 (91.7%) isolates belonging to the ST131 H89 strain (O16:H5) associated with both CTX-M-14 and CTX-M-24. Among the 37 non-ST131 isolates, 31 different sequence types were identified, precluding possible monoclonal spread. Among ST131 H30, the most common resistance genes were CTXM-15 (72.2%), OXA-1 (55.6%), and CTXM-27 (22.2%). Among ST131 non H30, the most common resistance genes were CTXM-14 (91.7%), CTXM-24 (91.7%), and TEM-1B (75.0%). Among non-ST131, the most common resistance genes were CTXM-15 (43.2%), TEM-1B (32.4%), OXA-1 (13.5%), and CTXM-14 (13.5%).

**Fig. 4:**
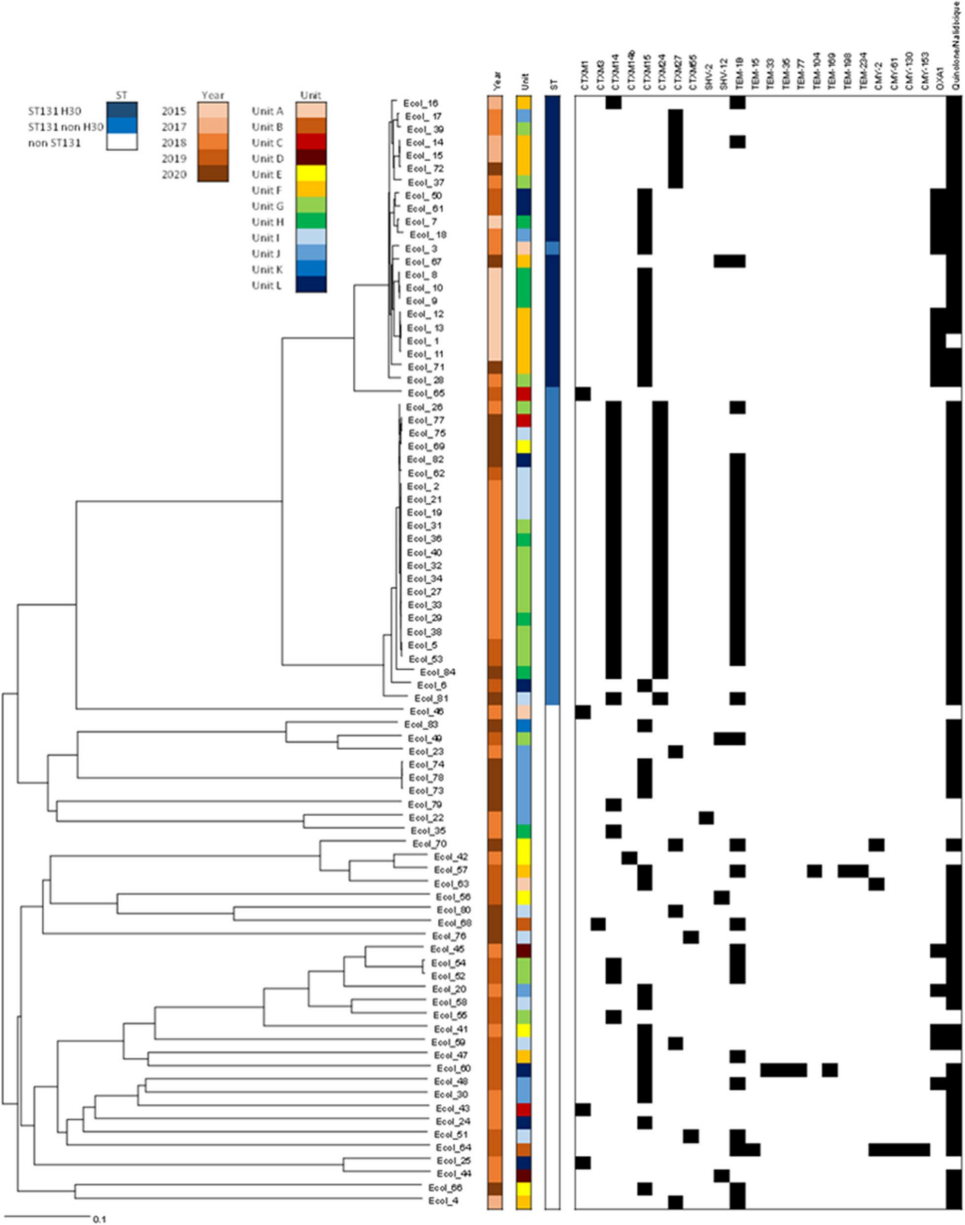
Dendrogram of sequenced ESBL-producing *Escherichia coli*, with epidemiological information and molecular data on ESBL genes; PCRH30 represent the results from the multi-array PCR, with non-ST131, ST131 H30, and ST131 non-H30

When considering epidemiological information from 2010 to 2020, we observed 27 nosocomial clusters of patients positive for ESBL-EC ST131 H30. Almost all (20/21) ESBL-EC ST131 H30 strains available for sequencing were genotypically related (Fig. 4). C2/H30-Rx strains dominated in 2015, while C1/H30-R was present in more recent clusters. Sixteen of these 21 (76%) strains were isolated from 2 wards (unit F and H) between 2015 and 2020. Twenty of 24 (83%) ST131 non-H30 strains available for sequencing were genotypically related and identified as ST131 H89 with the serotype O16:H5, which expressed the same CTX-M-14 and CTX-M-24 genes. We observed 5 clusters of patients positive for ST131 H89 in three wards (wards G, H, and I), with an attack rate of 12% (17 of 139 susceptible patients, Fig. 3). Finally, only 18% (5/27) of sequenced non-ST131 strains were genotypically related (Fig. 4).

## Discussion

The findings of these 11 yearly cross-sectional surveys support five main conclusions: (1) ESBL-EC prevalence increased over time in this university-affiliated LTCF, mainly driven by an increased proportion of previously known carriers; (2) after 2015, a decreasing prevalence of ST131 H30 subclones was observed over time, despite small localized outbreaks; (3) clonal expansion of ST131 H89 (O16:H5) subclones occurred since 2018, driven by multiple silent outbreaks; (4) We did not document the replacement by an emerging non-ST131 clone; and (5) no rebound effect in ESBL-EC or specific subclones was observed following discontinuation of contact precautions, though longer follow-up periods are needed to validate this finding.

LTCFs are well-known reservoirs for multiresistant ESBL-EC clones, with specific patient- and care-related exposures facilitating the spread of certain clades, including vulnerable and dependent patients with prolonged lengths of stay [24–29], as well as recognized challenges in implementing infection control measures [24, 28]. Many outbreaks report silent transmission of ESBL-EC ST131 in LTCF, especially belonging to the clade C2 (C2/H30-Rx-CTX-M-15) [1, 2, 6]. The rapid clonal expansion of this C2 clade through nosocomial outbreaks in LTCFs has already been observed to displace preexisting *E.coli* clades [6]. Thus, clonal fluctuance has been a recognized phenomenon with emergence and decline of temporarily successful clones. The persistence of certain *E. coli* clones, sporadically carrying carbapenemases genes, warrants further careful surveillance. [30]

Until now, few studies have reported nosocomial outbreaks associated with *E.coli* ST131 non-H30 clades. Population genomics on 4′071 global sources genomes observed a dominance of the clade C, co-circulating worldwide at stable frequencies [31]. In 2018, a single Spanish LTCF of 300 residents observed only 6 ST131 non-H30 associated with CTX-M-14 on 55 typed ESBL-EC isolates [32]. Our study observed that neither *E. coli* C2/H30-Rx, nor C1/H30-R, seem to drive the recent changing epidemiology of ESBL-EC in our LTCF, but rather a ST131 H89 harboring CTX-M-14 and CTX-M-27. This strain was sub-typed based on its *fimH* typing region, which is closely related and often associated to the H41 group of *E. coli* ST131 (1 SNP difference) [33–35]*.* To the best of our knowledge, there has been no outbreak report of this ST131 H89 *E. coli* subclone, which is anecdotally reported in population genomics [31, 35, 36].

The findings of this study are also in line with currently available evidence, which supports the discontinuation of contact precautions for ESBL-EC carriers, as suggested by European recommendations to limit ESBL-PE control measures in endemic settings to non-*E. coli* ESBL-PE [17]. Clonal outbreaks already occurred before discontinuation of contact precautions, and were not catalyzed by this decision. The observed recent clusters might not be an effect from lack of strict contact precautions, but rather direct consequences of the LTCF infrastructure, among other factors related to this specific setting [28, 37].

Though this study includes a large sample size and long-term surveillance data, we acknowledge the presence of several limitations. First, we could not quantify transmission and acquisition events due to the study design. Second, we acknowledge a potential bias in the selection of *E. coli* strains sequenced. Third, generalizability of our findings is impacted by the unicentric approach. Fourth, potential lack of genomic discrimination between highly similar *E. coli* clades was not possible due to the sequencing methods used (short reads). Fifth, the decreased length of stay could have impacted ESBL-EC prevalence by lowering the probability of nosocomial ESBL-EC acquisition and the proportion of known carriers. However, the acquisition risk in relation to the length of stay has been observed to be similar between ST131 and non-ST131 *E. coli*, and did not differ between 6 and 8 months of stay in LTCF. [12] Furthermore, ESBL-EC prevalence appears similar between LTCFs and among elderly living in the community. [38] For these reasons, the decreasing length of stay probably did not substantially influence ESBL-EC prevalence.

## Conclusion

The changing ESBL-EC epidemiology, emergence of novel clones, and related clusters in LTCF, though not impacted by discontinuation of contact precautions, should be monitored by a comprehensive screening and surveillance strategy.

## Supplementary Information

**Additional file 1.** Supplementary online content.

## Data Availability

The datasets used and/or analyzed during the current study are available from the corresponding author on reasonable request.
