# Supplementary material for: Epidemiology of ESBL-producing Escherichia coli from repeated prevalence studies over 11 years in a long-term-care facility
Source: Antimicrob Resist Infect Control. 2021 Oct 19;10:148. doi: 10.1186/s13756-021-01013-7 (PMC8527759; doi:10.1186/s13756-021-01013-7)
Supplement: Supplementary file 1 — Additional file 1. Supplementary online content. [file 13756_2021_1013_MOESM1_ESM.docx]

**Additional file 1. Supplementary Online Content**

Romain Martischang, Patrice François, Abdessalam Cherkaoui, Nadia Gaïa, Gesuele Renzi, Americo Agostinho, Monica Perez, Christophe E. Graf, Stephan Harbarth, et al. Epidemiology of ESBL-producing Escherichia coli from repeated prevalence studies over 11 years in a long-term-care facility.

**Figure S1**. Hand hygiene adherence of healthcare workers in long-term care facilities from 2014 to 2021

**Table S1.** Epidemiologic and genotypic characteristics of sequenced strains of ESBL-producing *Escherichia coli*

This appendix has been provided by the authors to provide readers additional information about this study.

**Additional file 1: Figures**

Figure S1. Hand Hygiene Adherence of Healthcare Workers in Long Term Care Facilities from 2014 to 2021


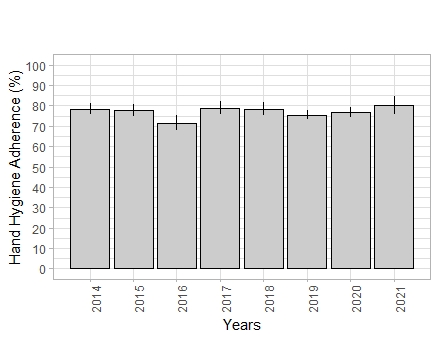


**Additional file 1: Tables**

Table S1. Epidemiologic and genotypic characteristics of sequenced strains of ESBL-producing Escherichia coli

| **Strain** | **Unite** | **Year** | **ST ^a^** | **PCR H30** | **fimH** | **Serotype** | **ESBL** | **Quinolone/Nalidixique** |
| --- | --- | --- | --- | --- | --- | --- | --- | --- |
| MR1 | F | 2015 | 131 | 1 |  |  | CTX-M-15;OXA-1 |  |
| MR2 | I | 2018 | 131 | 0 | 89 | H5O16 | CTX-M-14;CTX-M-24;TEM-1B | gyrA (p.S83L) |
| MR5 | G | 2019 | 131 | 0 | 89 | H5O16 | CTX-M-14;CTX-M-24;TEM-1B | gyrA (p.S83L) |
| MR6 | L | 2019 | 131 | 0 | 41 |  | CTX-M-15 | gyrA (p.S83L) |
| MR7 | H | 2015 | 131 | 1 |  |  | CTX-M-15;OXA-1 | gyrA (p.S83L), gyrA (p.D87N) |
| MR8 | H | 2015 | 131 | 1 |  |  | CTX-M-15 | gyrA (p.D87N), gyrA (p.S83L) |
| MR9 | H | 2015 | 131 | 1 |  |  | CTX-M-15 | gyrA (p.D87N), gyrA (p.S83L) |
| MR10 | H | 2015 | 131 | 1 |  |  | CTX-M-15 | gyrA (p.S83L), gyrA (p.D87N) |
| MR11 | F | 2015 | 131 | 1 |  |  | CTX-M-15;OXA-1 | gyrA (p.S83L), gyrA (p.D87N) |
| MR12 | F | 2015 | 131 | 1 |  |  | CTX-M-15;OXA-1 | gyrA (p.S83L), gyrA (p.D87N) |
| MR13 | F | 2015 | 131 | 1 |  |  | CTX-M-15;OXA-1 | gyrA (p.D87N), gyrA (p.S83L) |
| MR14 | F | 2017 | 131 | 1 |  |  | CTX-M-27;TEM-1B | gyrA (p.S83L), gyrA (p.D87N) |
| MR15 | F | 2017 | 131 | 1 |  |  | CTX-M-27 | gyrA (p.S83L), gyrA (p.D87N) |
| MR16 | F | 2017 | 131 | 1 |  |  | CTX-M-14;TEM-1B | gyrA (p.D87N), gyrA (p.S83L) |
| MR17 | J | 2018 | 131 | 1 |  |  | CTX-M-27 | gyrA (p.S83L), gyrA (p.D87N) |
| MR18 | J | 2018 | 131 | 1 |  |  | CTX-M-15;OXA-1 | gyrA (p.D87N), gyrA (p.S83L) |
| MR19 | I | 2018 | 131 | 0 | 89 | H5O16 | CTX-M-14;CTX-M-24;TEM-1B | gyrA (p.S83L) |
| MR20 | J | 2018 | 10 | 0 | 435 |  | CTX-M-15;OXA-1 | gyrA (p.D87N), gyrA (p.S83L) |
| MR21 | I | 2018 | 131 | 0 | 89 | H5O16 | CTX-M-14;CTX-M-24;TEM-1B | gyrA (p.S83L) |
| MR22 | J | 2018 | 73 | 0 | 10 |  | SHV-2 |  |
| MR23 | J | 2018 | 1193 | 0 | 64 |  | CTX-M-27 | gyrA (p.D87N), gyrA (p.S83L) |
| MR24 | L | 2018 | 410 | 0 | 24 |  | CTX-M-15 | gyrA (p.D87N), gyrA (p.S83L) |
| MR25 | L | 2018 | 5926 | 0 | 158 |  | CTX-M-1 |  |
| MR26 | G | 2018 | 131 | 0 | 89 | H5O16 | CTX-M-14;CTX-M-24;TEM-1B | gyrA (p.S83L) |
| MR27 | G | 2018 | 131 | 0 | 89 | H5O16 | CTX-M-14;CTX-M-24;TEM-1B | gyrA (p.S83L) |
| MR28 | G | 2018 | 131 | 1 |  |  | CTX-M-15;OXA-1 | gyrA (p.S83L), gyrA (p.D87N)/aac(6')-Ib-cr (aac(6')-Ib-cr_DQ303918) gyrA (p.S83L) |
| MR29 | H | 2018 | 131 | 0 | 89 | H5O16 | CTX-M-14;CTX-M-24;TEM-1B | gyrA (p.S83L) |
| MR30 | J | 2018 | 1431 | 0 | 32 |  | CTX-M-15 | gyrA (p.D87N), gyrA (p.S83L) |
| MR31 | G | 2018 | 131 | 0 | 89 | H5O16 | CTX-M-14;CTX-M-24;TEM-1B | gyrA (p.S83L) |
| MR32 | G | 2018 | 131 | 0 | 89 | H5O16 | CTX-M-14;CTX-M-24;TEM-1B | gyrA (p.S83L) |
| MR33 | G | 2018 | 131 | 0 | 89 | H5O16 | CTX-M-14;CTX-M-24;TEM-1B | gyrA (p.S83L) |
| MR34 | G | 2018 | 131 | 0 | 89 | H5O16 | CTX-M-14;CTX-M-24;TEM-1B | gyrA (p.S83L) |
| MR35 | H | 2018 | 104 | 0 | 2 |  | CTX-M-14 |  |
| MR36 | H | 2018 | 131 | 0 | 89 | H5O16 | CTX-M-14;CTX-M-24;TEM-1B | gyrA (p.S83L) |
| MR37 | G | 2018 | 131 | 1 |  |  | CTX-M-27 | gyrA (p.D87N), gyrA (p.S83L) |
| MR38 | G | 2018 | 131 | 0 | 89 | H5O16 | CTX-M-14;CTX-M-24;TEM-1B | gyrA (p.S83L) |
| MR39 | G | 2018 | 131 | 1 |  |  | CTX-M-27 | gyrA (p.D87N), gyrA (p.S83L) |
| MR40 | G | 2018 | 131 | 0 | 89 | H5O16 | CTX-M-14;CTX-M-24;TEM-1B | gyrA (p.S83L) |
| MR41 | E | 2018 | 617 | 0 | 29 |  | CTX-M-15;OXA-1 | gyrA (p.S83L), gyrA (p.D87N) |
| MR42 | E | 2018 | 38 | 0 | ND |  | CTX-M-14b |  |
| MR43 | C | 2018 | 224 | 0 | 61 |  | CTX-M-1 | gyrA (p.S83L), gyrA (p.D87N) |
| MR44 | D | 2018 | 57 | 0 | 27 |  | SHV-12 | gyrA (p.D87N), gyrA (p.S83L) |
| MR45 | D | 2018 | 10 | 0 | 54 |  | TEM-1B;OXA-1 | gyrA (p.D87N), gyrA (p.S83L) |
| MR46 | A | 2018 | 538 | 0 | 46 |  | CTX-M-1 |  |
| MR47 | F | 2019 | 8149 | 0 | ND |  | CTX-M-15;TEM-1B |  |
| MR48 | J | 2019 | 191 | 0 | 38 |  | CTX-M-15;TEM-1B;OXA-1 | qnrB1 (qnrB1_DQ351241), aac(6')-Ib-cr (aac(6')-Ib-cr_DQ303918) |
| MR49 | G | 2019 | 14 | 0 | 27 |  | SHV-12;TEM-1B | gyrA (p.S83L) |
| MR50 | L | 2019 | 131 | 1 |  |  | CTX-M-15;OXA-1 | aac(6')-Ib-cr (aac(6')-Ib-cr_DQ303918) gyrA (p.S83L) |
| MR51 | I | 2019 | 6448 | 0 | 60 |  | CTX-M-55;TEM-1B | gyrA (p.S83L), gyrA (p.D87N) |
| MR52 | G | 2019 | 167 | 0 | ND |  | CTX-M-14;TEM-1B | gyrA (p.D87N), gyrA (p.S83L) |
| MR53 | G | 2019 | 131 | 0 | 89 | H5O16 | CTX-M-14;CTX-M-24;TEM-1B | gyrA (p.S83L) |
| MR54 | G | 2019 | 167 | 0 | ND |  | CTX-M-14;TEM-1B | gyrA (p.D87N), gyrA (p.S83L) |
| MR55 | G | 2019 | 10 | 0 | 27 |  | CTX-M-14 |  |
| MR56 | E | 2019 | 925 | 0 | 54 |  | SHV-12 | qnrS1 (qnrS1_AB187515) |
| MR57 | F | 2019 | 38 | 0 | 5 |  | CTX-M-15;TEM-1B;TEM-104;TEM-198;TEM-234 | gyrA (p.S83L) |
| MR58 | I | 2019 | 3877 | 0 | 27 |  | CTX-M-15 | qnrS1 (qnrS1_AB187515) |
| MR59 | I | 2019 | 226 | 0 | 41 |  | CTX-M-15;CTX-M-27;OXA-1 | gyrA (p.S83L), gyrA (p.D87N)/qnrS13 (qnrS13_LUYD01000008) gyrA (p.S83L) |
| MR60 | L | 2019 | 46 | 0 | 34 |  | CTX-M-15;TEM-33;TEM-35;TEM-77;TEM-169 | qnrS1 (qnrS1_AB187515) |
| MR61 | L | 2019 | 131 | 1 |  |  | CTX-M-15;OXA-1 | aac(6')-Ib-cr (aac(6')-Ib-cr_DQ303918) gyrA (p.S83L) |
| MR62 | I | 2019 | 131 | 0 | 89 | H5O16 | CTX-M-14;CTX-M-24;TEM-1B | gyrA (p.S83L) |
| MR63 | A | 2019 | 3268 | 0 | 54 |  | CTX-M-15;CMY-2 | qnrS1 (qnrS1_AB187515) |
| MR64 | B | 2019 | 120 | 0 | 237 |  | TEM-1B;TEM-15;CMY-2;CMY-61;CMY-130;CMY-153 | qnrB19 (qnrB19_EU432277) |
| MR65 | C | 2019 | 131 | 0 | 22 | H4O25 | CTX-M-1 |  |
| MR66 | E | 2020 | 1722 | 0 | 153 |  | CTX-M-15;TEM-1B | qnrS1 (qnrS1_AB187515) |
| MR67 | F | 2020 | 131 | 1 |  |  | SHV-12;TEM-1B | gyrA (p.S83L), gyrA (p.D87N) |
| MR68 | B | 2020 | 1380 | 0 | 47 |  | CTX-M-3;TEM-1B | gyrA (p.S83L) |
| MR69 | E | 2020 | 131 | 0 | 89 | H5O16 | CTX-M-14;CTX-M-24 | gyrA (p.S83L) |
| MR70 | E | 2020 | 5150 | 0 | 65 |  | CTX-M-27;TEM-1B;CMY-2 | gyrA (p.S83L) |
| MR71 | F | 2020 | 131 | 1 |  |  | CTX-M-15;OXA-1 | gyrA (p.S83L), gyrA (p.D87N) - aac(6')-Ib-cr (aac(6')-Ib-cr_DQ303918) gyrA (p.S83L) |
| MR72 | F | 2020 | 131 | 1 |  |  | CTX-M-27 | gyrA (p.S83L), gyrA (p.D87N) |
| MR73 | J | 2020 | 636 | 0 | ND |  | CTX-M-15 | gyrA (p.S83L) |
| MR74 | J | 2020 | 636 | 0 | ND |  | CTX-M-15 | gyrA (p.S83L) |
| MR75 | I | 2020 | 131 | 0 | 89 | H5O16 | CTX-M-14;CTX-M-24 | gyrA (p.S83L) |
| MR76 | I | 2020 | 349 | 0 | 54 |  | CTX-M-55 | - |
| MR77 | C | 2020 | 131 | 0 | 89 | H5O16 | CTX-M-14;CTX-M-24 | gyrA (p.S83L) |
| MR78 | J | 2020 | 636 | 0 | ND |  | CTX-M-15 | gyrA (p.S83L) |
| MR79 | J | 2020 | 681 | 0 | 3 |  | CTX-M-14 |  |
| MR80 | I | 2020 | 69 | 0 | 27 |  | CTX-M-27 | gyrA (p.S83L) |
| MR81 | I | 2020 | 131 | 0 | 89 | HXO16 | CTX-M-14;CTX-M-24;TEM-1B | gyrA;;bvzc (p.S83L) |
| MR82 | L | 2020 | 131 | 0 | 89 | H5O16 | CTX-M-14;CTX-M-24;TEM-1B | gyrA (p.S83L) |
| MR83 | K | 2020 | 1446 | 0 | 30 |  | CTX-M-15 | qnrS1 (qnrS1_AB187515) |
| MR84 | H | 2020 | 131 | 0 | 89 | H5O16 | CTX-M-14;CTX-M-24 | gyrA (p.S83L) |

Footnote to Additional file 1: Table S1.

^a^ Sequence types were determined based on the allelic discrimination qPCR assays described in the methods to ascertain ST131 lineages, and based on MLST for negative ST131 results.
